# The dissociating effects of fear and disgust on multisensory integration in autism: evidence from evoked potentials

**DOI:** 10.3389/fnins.2024.1390696

**Published:** 2024-08-05

**Authors:** Maria Elena Stefanou, Neil M. Dundon, Patricia E. G. Bestelmeyer, Monica Biscaldi, Nikolaos Smyrnis, Christoph Klein

**Affiliations:** ^1^Department of Child and Adolescent Psychiatry, Psychotherapy, and Psychosomatics, Medical Center, University of Freiburg, Freiburg, Germany; ^2^Department of Neuroimaging, Institute of Psychiatry, Psychology and Neuroscience, King’s College London, London, United Kingdom; ^3^Department of Psychological and Brain Sciences, University of California, Santa Barbara, Santa Barbara, CA, United States; ^4^Department of Psychology, Bangor University, Bangor, United Kingdom; ^5^Second Department of Psychiatry, National and Kapodistrian University of Athens, Medical School, University General Hospital “Attikon”, Athens, Greece; ^6^Laboratory of Cognitive Neuroscience and Sensorimotor Control, University Mental Health, Neurosciences and Precision Medicine Research Institute “COSTAS STEFANIS”, Athens, Greece; ^7^Department of Child and Adolescent Psychiatry, Medical Faculty, University of Cologne, Cologne, Germany

**Keywords:** autism spectrum disorders, emotion perception, multisensory integration, Miller’s Race Model, EEG, dynamic stimuli

## Abstract

**Background:**

Deficits in Multisensory Integration (MSI) in ASD have been reported repeatedly and have been suggested to be caused by altered long-range connectivity. Here we investigate behavioral and ERP correlates of MSI in ASD using ecologically valid videos of emotional expressions.

**Methods:**

In the present study, we set out to investigate the electrophysiological correlates of audiovisual MSI in young autistic and neurotypical adolescents. We employed dynamic stimuli of high ecological validity (500 ms clips produced by actors) that depicted fear or disgust in unimodal (visual and auditory), and bimodal (audiovisual) conditions.

**Results:**

We report robust MSI effects at both the behavioral and electrophysiological levels and pronounced differences between autistic and neurotypical participants. Specifically, neurotypical controls showed robust behavioral MSI for both emotions as seen through a significant speed-up of bimodal response time (RT), confirmed by Miller’s Race Model Inequality (RMI), with greater MSI effects for fear than disgust. Adolescents with ASD, by contrast, showed behavioral MSI only for fear. At the electrophysiological level, the bimodal condition as compared to the unimodal conditions reduced the amplitudes of the visual P100 and auditory P200 and increased the amplitude of the visual N170 regardless of group. Furthermore, a cluster-based analysis across all electrodes revealed that adolescents with ASD showed an overall delayed and spatially constrained MSI effect compared to controls.

**Conclusion:**

Given that the variables we measured reflect attention, our findings suggest that MSI can be modulated by the differential effects on attention that fear and disgust produce. We also argue that the MSI deficits seen in autistic individuals can be compensated for at later processing stages by (a) the attention-orienting effects of fear, at the behavioral level, and (b) at the electrophysiological level via increased *attentional effort*.

## Highlights

- MSI is key for everyday interactions and has been implicated in the social deficits reported in ASD.
- Previous research has reported MSI deficits in autistic individuals through various paradigms. However, findings have been inconsistent, especially regarding to whether MSI is facilitated independently of attention.
- Most studies investigating MSI in ASD have either done so with non-social stimuli or have used stimuli of low-ecological validity.
- To our knowledge, we are the first study to combine highly ecologically valid stimuli with EEG where we report MSI deficits in autistic adolescents, for which they compensate via attentional mechanisms.
- In this context, a corroboration of these findings from future research could lead to the development of techniques to improve MSI and thus higher-level deficits.

## 1 Introduction

Deficits in social interaction and communication are among the key characteristics of Autism Spectrum Disorder (ASD) and have been the topic of a wealth of ASD research. In addition, sensory abnormalities—often reported by clinicians to characterize the experiences of autistic individuals—have become increasingly acknowledged as a central dysfunction of the disorder (Klein et al., 2022) and are now featured in the DSM-5 as one of the “B criteria” (American Psychiatric Association, 2013). And indeed, various studies have reported altered sensory processing in a broad range of sensory domains (Gomot et al., 2008; Kujala et al., 2013). While the subjective sensory experience of autistic individuals is often characterized by hyper-sensitivity to auditory, visual or tactile stimuli (Klein et al., 2022), objective measures of abnormal sensory experience have pointed to problems in integrating information from different sensory channels, such as Multisensory Integration (MSI; Iarocci et al., 2010; Brandwein et al., 2013; Collignon et al., 2013; De Boer-Schellekens et al., 2013; Woynaroski et al., 2013; Katagiri et al., 2014; Stevenson et al., 2014). MSI refers to the integration of information conveyed simultaneously through different sensory channels (e.g., seeing and hearing another person speaking). MSI processes begin very early in sensory information processing (Stefanou et al., 2019). They are considered automatic and unconscious (Meredith and Stein, 1983, 1986; Stein et al., 2014) and lead to more accurate and speeded recognition of the incoming information (Giard and Peronnet, 1999; Brandwein et al., 2011), in particular if sensory information is noisy, for example during a cocktail party (Kayser and Logothetis, 2007). Regarding humans, the efficient integration of information conveyed by the face and voice (moving lips and spoken words) is key to efficient communication (Gervais et al., 2004; Magnée et al., 2011). The well-known McGurk effect (McGurk and MacDonald, 1976) underlines this argument and suggests that MSI processes are automatic and not under voluntary control. However, results on the potential role of attention on MSI still remain contradictory (Hartcher-O’Brien et al., 2017; but see also Koelewijn et al., 2010 for the effects of attention and the stages of MSI).

With regards to the effects of attention more specifically, some studies suggest that MSI occurs automatically (regardless of attention), while others suggest that top-down attention influences successful MSI (Fernández et al., 2015). The complexity of the MSI—attention interplay can be seen by studies suggesting that any attention effects on MSI differ depending on the stage of sensory processing, that is, at the early MSI stage we integrate information pre-attentively whereas at the later MSI stage attention is necessary for successful MSI to be facilitated (Koelewijn et al., 2010; Stefanou et al., 2019). Such findings have left attention’s role on MSI not well understood and a matter of debate (Hartcher-O’Brien et al., 2017).

Impaired MSI in ASD has been demonstrated using various paradigms, including simple RT tasks (Brandwein et al., 2013), multisensory speech paradigms (Foxe et al., 2015), cross-modal temporal order judgment tasks (Kwakye et al., 2011), multisensory illusion tasks (Mongillo et al., 2008; Foss-Feig et al., 2010; Woynaroski et al., 2013; Bebko et al., 2014; Stevenson et al., 2014) and visual search tasks (Collignon et al., 2008). These paradigms have conjointly demonstrated the multiple facets of the MSI deficit in ASD. Among these paradigms are tasks like the one developed by Charbonneau et al. (2013) that trigger MSI processes for the recognition of emotions using ecologically valid video-recorded emotional exclamations of the six basic emotions by professional actors (Simon et al., 2008). More specifically, Charbonneau et al. (2013) demonstrated the behavioral effects of MSI employing emotional exclamations of disgust and fear, which were selected due to their biological significance. The results showed that autistic participants exhibited decreased emotion recognition across visual and auditory modalities and reduced or absent behavioral MSI. Notably, from an evolutionary perspective fear enhances attention to alert of a potentially threatening situation while disgust diverts it to reduce exposure (Susskind et al., 2008; Krusemark and Li, 2011) while in previous studies of our lab we have confirmed that the dissociating effects of fear and disgust on attention can extent to MSI and facilitate it or diminish it (Stefanou et al., 2019).

As mentioned before, MSI occurs very early in sensory processing and seems to be triggered automatically (but see also Koelewijn et al., 2010; Stefanou et al., 2019, 2020 for the effects of attention in later processing stages) as shown in the superior colliculus of anaesthetized cats (Meredith and Stein, 1983). Likewise, Event-Related Potential (ERP) studies, taking advantage of the excellent temporal resolution of the EEG technology, have supported that cortical MSI effects begin early in information processing. Electro-cortical MSI effects are expressed as a distinct activation pattern when comparing the amplitude of the bimodal condition and the sum of the unimodal ones; this distinct activation can be seen either as increased or decreased activity that cannot be explained by the sum of the unimodal conditions [AV – (A + V)], called super-additivity and sub-additivity, respectively. Typically, these MSI effects are found as early as 40 and 90 ms after stimulus onset over various scalp areas, both in adults and in children (Giard and Peronnet, 1999; Molholm et al., 2002; Brett-green et al., 2008; Brandwein et al., 2011; Stefanou et al., 2020). MSI effects are also seen through increased amplitudes of visual components such the P100 and N170 (Stefanou et al., 2019, 2020) when comparing the bimodal with unimodal conditions and compared to the sum during these components’ time-windows (McCracken et al., 2019). In terms of auditory components, studies also typically report reduced amplitudes in the bimodal conditions for the N100 (Magnée et al., 2008; Jessen and Kotz, 2011) and increased amplitude for the P200 (Jessen and Kotz, 2011; but see also Magnée et al., 2011 that reported smaller P200 amplitudes). With regards to latency-specific effects, studies report faster latencies for the auditory N1 and P2 components (Magnée et al., 2009) and visual N100 latency (Stefanou et al., 2020).

In autistic individuals, evidence of altered MSI stems from differing neurophysiological findings. Firstly, analysis of MSI effects across various scalp areas have been reported to be delayed compared to controls (Russo et al., 2010; Stefanou et al., 2020; Dwyer et al., 2022). Secondly, in terms of amplitudes, autistic individuals show overall reduced MSI effects compared to controls over central areas (Dwyer et al., 2022). Finally, symptom severity has been found to correlate significantly with amplitude-related MSI effects in autistic individuals with these effects being decreased for individuals with severe symptoms compared to controls, and individuals with moderate symptoms lying in between of these two groups (Brandwein et al., 2015).

Another important aspect of studying MSI is the ecological validity of the stimuli given that MSI is crucial for perceiving social situations and emotions (Young and Bruce, 2011; Skuk and Schweinberger, 2013; Thye et al., 2018). The role of verbal and facial expression of affect is one domain of communication that plays a pivotal role in functioning social interaction. The perception and recognition of all basic emotions (Darwin, 1872, 2009) is considered inborn (Walker-Andrews, 1998) and entails the efficient integration of face and voice. That emotion perception is indeed multimodal, has been found in various studies (Young and Bruce, 2011; Skuk and Schweinberger, 2013). MSI is thus an essential process of our everyday life, necessary for successful social interaction (Magnée et al., 2011). And yet, the majority of the studies investigating the MSI of emotions have neglected this in one or the other way. Specifically, studies investigating MSI and emotion perception in ASD have typically used stimuli with low ecological validity such as black and white still pictures superimposed with sound (for example, Magnée et al., 2008). Ecological validity should be a requirement for both behavioral and neurophysiological studies but so far studies using stimuli with high ecological validity are mainly behavioral studies (for example, Charbonneau et al., 2013) or limited to healthy adults (for example, Jessen and Kotz, 2011; Skuk and Schweinberger, 2013; Stefanou et al., 2019).

Our study aimed to explore MSI in young adolescents with ASD using ecologically valid, dynamic emotional stimuli. We aimed to replicate the behavioral results of Charbonneau et al. (2013) and to explore the neurophysiological bases of MSI with the use of ERPs. We expected that children with ASD would show a decreased benefit from the bimodal (audiovisual) compared to the unimodal (auditory vs. visual) presentation of emotions, compared to neurotypical controls. We also expected the bimodal condition to lead to an increase in the amplitude of the visual components, an amplitude decrease specific to the auditory components, and for these effects to be stronger for controls than adolescents with ASD. We also aimed to investigate the spatio-temporal evolution of MSI and expected robust effects starting early on after stimulus presentation *for neurotypical controls* with a delayed MSI effect and limited scalp distribution for *adolescents with ASD*.

## 2 Methods

The following experimental protocol was approved by the Ethics Committee of the Albert Ludwigs-University of Freiburg (ethics vote no. 238/15) and all participants’ information was treated according to the declaration of Helsinki. Participants and their parents/legal guardians provided informed written consent, after receiving a verbal and written description of the study.

### 2.1 Participants

A total of 50 children (21 with ASD; 29 controls) between the ages of 11 and 14 years were recruited through the database of the Clinic for Child and Adolescent Psychiatry, Psychotherapy, and Psychosomatics of the University of Freiburg as part of a larger study (Stefanou et al., 2020).

Participants with autism were selected based on already having received the F84.0, F84.1 or F84.5 diagnoses according to the International Classification of Diseases (ICD-10; World Health Organisation, 1992), as given by an experienced psychiatrist/psychologist of the Clinic. Diagnoses were based on anamnestic interviews with parents and children, the administration of the German version of Autism Diagnostic Observation Schedules (Rühl et al., 2004) and the Autism Diagnostic Interview-Revised (Bölte et al., 2005). ADOS-2 (Poustka et al., 2015) was used with four participants.

All participants with autism except two were medication-free. One participant showing ADHD symptoms but not fulfilling the ADHD diagnostic criteria was given methylphenidate to reduce irritability and inattention in social situations. This participant was medication-free during the testing (paused treatment 24 h prior to the testing sessions). Another autistic participant was receiving antipsychotics (Abilify) due to a comorbid diagnosis of obsessive-compulsive disorder.

Exclusion criteria for both groups were a first language other than German, comorbid diagnoses such as motor ticks, epilepsy, ADHD, or an IQ score below 70 (as assessed with the Cultural Fair Intelligence Test 20-R, CFT 20-R; Weiß and Weiß, 2006). Furthermore, participants were excluded from data analysis if EEG data were heavily contaminated by artifacts such as muscle or movement artifacts, or if they failed or refused to complete at least five blocks (~83%) out of the six blocks of the task. For healthy children, scores outside the Social Responsiveness Scale (SRS; Constantino and Gruber, 2005) normal range was also an exclusion criterion.

After the application of the exclusion criteria, the final sample consisted of 15 children with ASD (10 male, 14 right-handed) and 25 healthy children (13 male, 22 right-handed; see Table 1). Participants were compensated for their time with one cinema or book voucher (worth 7.50 €) per hour.

**Table 1:** Group characteristics and scores.

	*TDs (25)*		*ASD (15)*		*t-test*	
	Mean *(SD)*	Range	Mean *(SD)*	Range	*t*	*p*-values
Age	13.0 *(0.90)*	11.41–14.62	13.03 *(0.89)*	11.61–14.81	0.084	0.934
IQ^*^	127.72 *(16.2)*	93–154	97.87 *(18.58)*	70–140	−5.341	***<0.001***
SRS raw *(N = 14)*	14.04 *(15.92)*	0–73	83.4 *(37.63)*	0–131	8.134	***<0.001***
SRS T-norms	39.92 *(13.17)*	23–74	78.07 *(11.06)*	56–92	9.189	***<0.001***
ADOS-G/ADOS II	*N.A.*	*N.A.*	12.15/9 *(4.81/9.9)*	6–22/2–16	__	__
ADI-R *(N = 14)*	*N.A.*	*N.A.*	15.57 *(5.83)*	3–25	__	__
Social interaction						
ADI-R *(N = 14)*	*N.A.*	*N.A.*	11.21 *(4.77)*	3–18	__	__
Communication						
ADI-R *(N = 14)*	*N.A.*	*N.A.*	4.57 *(2.88)*	0–9	__	__
Restricted, repetitive, and stereotyped behaviors and interests						
ADI-R *(N = 14)*	*N.A.*	*N.A.*	2.29 *(1.73)*	0–5	__	__
Abnormal development until 3 years of age						

^*^IQ as measured with the Cultural Fair Intelligence Test (CFT 20-Revised). *p*-values in bold represent statistically significant results.

### 2.2 Stimuli and procedure

The current experiment was part of a larger study and was completed over three sessions (see Stefanou et al., 2020), with the first two sessions being dedicated for the completion of two MSI tasks with simultaneous EEG recording and the third one for the administration of the CFT 20-R (Weiß and Weiß, 2006). We here present results from children completing a two-forced-choice emotion recognition task presented using Psychophysics Toolbox extensions 3.0.12 (Brainard, 1997; Pelli, 1997; Kleiner et al., 2007) on MATLAB software (R2015a; The MathWorks, Inc., Natick, Massachusetts, United States). The two presented emotions were fear and disgust, as in Charbonneau et al. (2013) and were presented by four actors (2 female, 2 male) in an auditory (vocalizations), visual (video without sound) and bimodal (video with sound) condition. Each trial contained a pre-stimulus interval of 2,000–3,000 ms (pooled from an exponential distribution with a mean of ~2,400 ms), followed by a 500 ms stimulus presentation tuned to the screen’s refresh rate, and a response period of 2,000 ms. A white fixation cross was present during the pre-stimulus interval, the response period and the auditory condition. The task comprised of six blocks with each block containing 120 trials (720 trials in total; 120 per emotion per condition). All conditions were interspersed within each block in a pseudorandom order. Stimuli were dynamic videos adapted from Simon et al. (2008) and were processed with Adobe Premiere Elements (Adobe Systems, Inc.). To match the original stimuli by Charbonneau et al. (2013), and as described in Stefanou et al. (2019), video stimuli were segmented to sequences of 500 ms (15 frames) and the audio clips were exported based on these sequences. Subsequently, we ensured that all stimuli started with a *neutral expression lasting for 1 frame* and evolved into full expression afterwards. Given that visual dominance (that is, more efficient visual than auditory processing) was evidenced in previous studies with such stimuli (Collignon et al., 2008; Stefanou et al., 2019) we reduced the reliability of stimuli in order to account for modality dominance. For this, we parsed the six blocks of trials into two sets of three. In one set, we degraded stimuli by adding uniform white noise to the visual channel of visual unimodal and bimodal conditions, at each frame, pixel and the three color dimensions (RGB). In the other set, we added noise to the auditory channels of all auditory unimodal and bimodal conditions; audio files were first normalized to the range [−1 1] while uniform noise was added to the matrix of the raw audio wave and presented at 75 dB SPL. This ensured that any differences between the unimodal and bimodal conditions were due to MSI effects, and not due to the white noise being presented only in the bimodal condition. Each modality of noise was calibrated using a threshold task to establish individually tailored accuracy levels of 80%. This calibration was achieved by degrading the unimodal stimuli in eight levels of noise (60%–95% vs. 40%–75% for visual noise and 50%–85% vs. 40%–75% for auditory noise for controls and patients, respectively, and in increments of 5%).

These individually tailored noise levels were subsequently used for the main task (visual noise for controls: 76.12 ± 8.5%, for ASD: 67.73 ± 17.43%; controls vs. ASD: *p* = 0.048; auditory noise for controls: 71.92 ± 12.47%; for ASD: 64.8 ± 20.79%; controls vs. ASD: *p* = 0.182). Consistent with previous studies using a similar approach (Charbonneau et al., 2013) controls showed a significantly higher noise threshold than patients only for the visual modality [*F*_(1,38)_ = 4.183, *p* = 0.048].

Brain Vision Recorder (Brain Products, Gilching), two BrainAmps DC amplifiers and a 64-channel actiCap (Brain Products, Gilching) were used for the acquisition of EEG according to the International 10–10System (American Electroencephalographic Society, 1991). The EEG was recorded with a 500 Hz sampling rate, with impedances kept below 5kΩ. FCz and AFz electrodes served as reference and ground, respectively. Finally, two infraorbital channels were placed vertically under each eye, and an additional electrode was positioned at the Nasion.

### 2.3 Behavioral data analysis

Responses were defined as the first response that accurately identified the presented emotion within 150 and 1,800 ms relative to stimulus onset. Median reaction times (RT) and the standard variability of responses (SDRT) were calculated for correct responses. Median RT, SDRT, and accuracy were submitted to two separate 2*2*2 mixed model ANOVAs, one for trials degraded with visual noise, and a second one for trials degraded with auditory noise. Both ANOVAs included CONDITION [unimodal (visual/auditory), bimodal (audiovisual)] and EMOTION (fear, disgust) as within-subjects factors and GROUP (ASD, controls) as the between-subjects factor.

In order to evaluate whether any decrease in RT was due to MSI, we applied Miller’s Race Model Inequality (RMI; Miller, 1982) using Matlab (as described in Ulrich et al., 2007). The procedure used in this study was identical to the one described in Stefanou et al. (2019). Miller’s RMI was calculated at every 5^th^ percentile for each participant using the RT distributions of the auditory, visual and bimodal conditions. The “bound,” that is, the bimodal RT distribution hypothesized under Miller’s Race Model, was also calculated at every 5th percentile of the distribution (5th–100th percentile) across emotions, as well as for each emotion separately.

### 2.4 EEG processing and analyses

EEG data were processed offline using Brain Vision Analyzer (Version 2.0, Brain Products, Gilching). Data were down-sampled to 100 Hz and filtered with a 0.1–40 Hz bandpass filter. During a first data inspection, any data sections with a voltage of ≤0.5 μV or ≥ 1,500 μV for a duration of 200 ms or more were marked as artifact-contaminated and were excluded from further analysis. Data were segmented into 1,900 ms epochs (−200 ms to 1,700 ms relative to stimulus onset). Segments were then submitted to an Infomax Independent Component Analysis (ICA) and components representing eye blinks, saccades, and muscle activity were manually identified and removed by not back-projecting them to the electrode space through a semi-automatic ICA Inverse. A second data inspection was performed and segments with activity ≤0.5 or ≥ 200 μV for a duration of ≥200 ms were again excluded. After re-referencing to the common average reference, data were concatenated across sessions, re-grouped according to condition, emotion and whether trials were degraded with visual or auditory noise. The baseline was then normalized to the period of 200 ms pre-stimulus onset, and segments containing only correct responses were averaged for each participant. This resulted in retaining a minimum of 67% (M = 82; SD = 7%) of the trials of interest for controls and a minimum of 58% (M = 76%; SD = 12%) for patients.

#### 2.4.1 Sensory and perceptual event-related potentials

The auditory N100 and P200, the visual P100 and N170 and the late positive component (LPC) were identified through visual inspection of both the grand and the individual averages. The peaks for each component were determined separately for each subject, emotion and condition, as the maximum peak in a defined electrode channel and a defined interval. The auditory N100 component was identified at central electrodes C1, Cz, C2 between 70 and 150 ms after stimulus-onset, and the auditory P200 again at electrodes C1, Cz, C2 between 170 and 250 ms. The visual P100 component was identified at occipital electrodes (O1, Iz, O2) between 80 and 180 ms, while the visual N170 was identified at parietal and temporal–parietal electrodes between 170 and 260 ms (P2, TP8, P8). Given the large deflection of the P100, the ongoing negativity of the N170 remained of positive amplitude at the expected occipital-parietal (e.g., Batty and Taylor, 2003) and temporal-occipital electrodes. Given that several studies investigate the maxima of N170 in lateral posterior electrodes (see, Eimer and Holmes, 2003; Almeida et al., 2016), we chose the aforementioned temporal–parietal electrodes where N170 was most prominent. LPC was identified at electrodes Pz and POz between 350 and 850 ms for all three conditions.

Peak detection for the auditory N100 and P200, and the visual P100 and N170, was performed in a semi-automatic mode in order to visually inspect the peaks and detect possible variance between participants, and the mean amplitude in a ± 10 ms window around the peak of each component was exported. For the LPC, due to the broad nature of this component, we exported the mean area activity between the 350 and 850 ms time window. In order to investigate MSI interactions, the sum of the unimodal conditions [auditory + visual] and difference waves (bimodal – [auditory + visual]) were also calculated.

All subsequent ANOVAs included GROUP (ASD vs. controls) as the between-subjects factor. Amplitudes and latencies of the visual P100 were submitted to a 2*2*2*3 mixed-model ANOVA with CONDITION (visual, bimodal), EMOTION (fear, disgust) and ELECTRODE (O1, Iz, O2) as within-subjects factors. Likewise, N170 amplitudes and latencies were submitted to a 2*2*2*3 mixed-model ANOVA with CONDITION (visual, bimodal), EMOTION (fear, disgust) and ELECTRODE (P2, TP8, P8) as within-subjects factors. Two separate 2*2*2*3 mixed-model ANOVAs were performed for the auditory N100 and P200 amplitudes and latencies, with CONDITION (auditory, bimodal), EMOTION (fear, disgust) and ELECTRODE (C2, Cz, C1) as within-subjects factors. LPC area activity was submitted to two separate 2*2*2 ANOVAs, one for trials degraded with visual noise and one for trials degraded with auditory noise, with CONDITION (bimodal, unimodal), EMOTION (fear, disgust) and ELECTRODE (Pz, POz) as within-subjects factors. Due to the broad peak of this component, latencies were not analyzed. Any significant interactions revealed from the ANOVAs described in this section were submitted to post-hoc analyses (see corresponding results sections).

#### 2.4.2 Evolution of multisensory integration

The sum of the two unimodal conditions cannot be equalized with the bimodal condition since it is missing the pure MSI effect (for a review, see Stein and Stanford, 2008). For this reason, we assessed the spatio-temporal course of MSI by contrasting the bimodal and sum conditions through a cluster-based permutation test using the Monte Carlo method, a non-parametric analysis implemented in the Fieldtrip toolbox (Maris and Oostenveld, 2007; Maris, 2012; please see Stefanou et al., 2020 for more details). The analysis was also performed for the two levels of the EMOTION factor.

### 2.5 Analysis of covariance

Given that patients and controls differed significantly in IQ, we performed additional ANCOVAs using IQ as a covariate for both the behavioral and the ERP data. Results were robust regarding IQ unless mentioned otherwise in the corresponding results section.

## 3 Results

### 3.1 Behavioral results

#### 3.1.1 Median RT

##### 3.1.1.1 Bimodal vs. auditory

Fear produced overall faster responses than disgust [EMOTION: *F*_(1,38)_ = 7.97, *p* = 0.008, η_p_^2^ = 0.173]. The bimodal condition also produced a significant speed-up of RT compared to the auditory [CONDITION: *F*_(1,38)_ = 90.34, *p* < 0.001, η_p_^2^ = 0.703] suggesting an MSI-related speed-up. This MSI-related speed-up seen in the bimodal compared to the *auditory condition*, was greater for trials presenting disgust [−215.58 ms; *t*_(39)_ = −8.734, *p* < 0.001] than trials presenting fear [−165.94 ms; *t*_(39)_ = −9.510, *p* < 0.001; CONDITION*EMOTION: *F*_(1,38)_ = 6.500, *p* = 0.015, η_p_^2^ = 0.146].

##### 3.1.1.2 Bimodal vs. visual

There was again a main effect of fear producing overall faster responses than disgust [EMOTION: *F*_(1,38)_ = 27.958, *p* < 0.001, η_p_^2^ = 0.424] and a main effect of condition with the bimodal condition producing faster RT compared to the visual condition [CONDITION: *F*_(1,38)_ = 61.507, *p* < 0.001, η_p_^2^ = 0.618]. When comparing the bimodal and the *visual condition*, the MSI-related speed-up was further increased for trials of fear [−84.82 ms; *t*_(39)_ = −8.411, *p* < 0.001] than for disgust [−57.263 ms; *t*_(39)_ = −6.064, *p* < 0.001; CONDITION*EMOTION: *F*_(1,38)_ = 8.398, *p* = 0.006, η_p_^2^ = 0.181].

#### 3.1.2 Variability of responses

Participants responded significantly less variably (measured by SDRT) during the bimodal compared to the auditory [CONDITION in Bimodal vs. Auditory: *F*_(1,38)_ = 56.427, *p* < 0.001, η_p_^2^ = 0.598] and visual condition [CONDITION in Bimodal vs. Visual: *F*_(1,38)_ = 9.67, *p* = 0.003, η_p_^2^ = 0.205]. No other effects, interactions or group differences were found.

#### 3.1.3 Accuracy

##### 3.1.3.1 Bimodal vs. auditory

Participants responded overall more accurately during the bimodal compared to the auditory condition [CONDITION: *F*_(1,38)_ = 6.063, *p* < 0.001, η_p_^2^ = 0.612] suggesting improved recognition due to MSI. This MSI-related improved recognition seen in the bimodal compared to the *auditory condition* was amplified for trials of disgust [20.4%; *t*_(39)_ = −6.456, *p* < 0.001] rather than fear [12.1%; *t*_(39)_ = 5.369, *p* < 0.001; CONDITION*EMOTION: *F*_(1,38)_ = 4.869, *p* = 0.033, η_p_^2^ = 0.114].

##### 3.1.3.2 Bimodal vs. visual

There was again a main effect of emotion and of condition where participants responded more accurately during fear than disgust and during the bimodal compared to the visual condition [EMOTION: *F*_(1,38)_ = 7.292, *p* = 0.010, η_p_^2^ = 0.161; CONDITION: *F*_(1,38)_ = 34.787, *p* < 0.001, η_p_^2^ = 0.478]. On the contrary, and similarly to RTs, when comparing the bimodal and the *visual condition*, accuracy improvement was significantly increased for the emotion of fear [8.2%0.121; *t*_(39)_ = 5.899, *p* < 0.001] than for disgust [3.9%; *t*_(39)_ = 3.219, *p* = 0.001; CONDITION*EMOTION: *F*_(1,38)_ = 6.462, *p* = 0.015, η_p_^2^ = 0.145]. No significant effect of GROUP or interaction with the factor GROUP was found in any of the above contrasts.

Overall, the *key* behavioral results show that all participants showed faster RTs, increased accuracy and smaller ISVs (see Table 2; Supplementary Table 1), for the bimodal compared to the unimodal conditions. However, the behavioral improvement during the bimodal condition was amplified by fear when compared to the visual condition contrasts and by disgust when compared to the auditory condition.

**Table 2:** Descriptive statistics of behavioral responses.

	Controls				ASD			
	A	AV_A	AV_V	V	A	AV_A	AV_V	V
**Median RT (ms)**								
*Fear*	897.58 (32.66)	707.05 (25.19)	676 (21.19)	761.22 (23.03)	846.73 (42.16)	721.03 (32.51)	723.17 (27.35)	807.33 (29.73)
*Disgust*	945.3 (40.01)	712.76 (20.64)	746.02 (22.77)	804.16 (24.83)	912.33 (51.65)	725.03 (26.65)	767.43 (29.39)	823.23 (32.05)
**SDRT (ms)**								
*Fear*	199.83 (11.78)	161.43 (11.36)	164.32 (10.78)	179.55 (11.28)	210.64 (15.20)	166.81 (14.67)	171.27 (13.92)	183.22 (14.57)
*Disgust*	203.86 (10.78)	154.22 (9.41)	155.64 (10.05)	168.38 (10.79)	219.79 (13.79)	165.59 (12.20)	165.23 (12.98)	187.64 (13.93)
**Accuracy (%)**								
*Fear*	75.42 (3)	86.67 (2.1)	91.03 (1.5)	82.70 (2.6)	71.17 (3.9)	84.56 (2.7)	87.39 (1.9)	79.39 (3.3)
*Disgust*	71.42 (4.1)	90.95 (1.5)	93.3 (1.4)	88.03 (2.1)	67.06 (5.3)	89.06 (1.9)	89.83 (1.8)	88.17 (2.7)

SE, the standard error of the mean is denoted in parenthesis; A, the auditory condition; V, the visual condition; AV_A, the bimodal condition with auditory degradation; and AV_V, the bimodal condition with visual degradation.

#### 3.1.4 Miller’s Race Model Inequality

**Figure 1:**
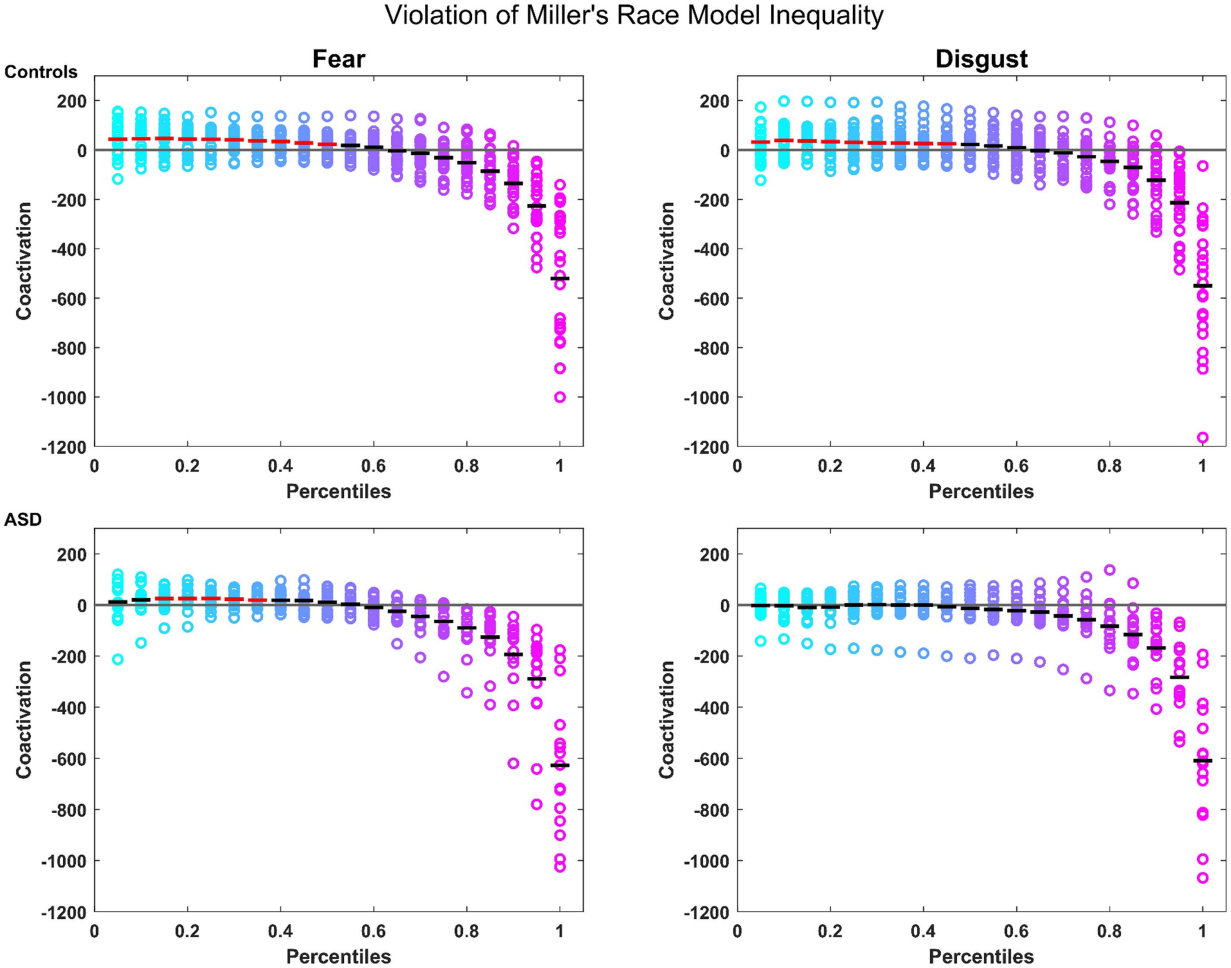
Miller’s Race Model Inequality (RMI): for controls (top row) and patients (bottom row) for the emotions of fear and disgust. Colored circles represent the coactivation for individual participants across each percentile. The mean is represented by the horizontal bars in black if there was no violation of the model, and in red where the violation was statistically significant. The horizontal gray line represents the bound of the model.

Notably, controls presented a robust MSI that was significant from the 5th to the 45th percentile where patients failed to show any significant MSI. When split by emotions, controls showed again a robust MSI for both emotions (significant violation of the model from the 5th to the 50th percentile for fear and from the 5th to the 45th percentiles for disgust; see Figure 1). Conversely, patients showed a violation of the model *only* for the emotion of fear, and *only* from the 15th to the 35th percentile (see Figure 1). These findings confirm the reported MSI deficits in autistic individuals, for which they can potentially compensate through the attentional mechanisms associated with fear.

### 3.2 EEG results

#### 3.2.1 Visual P100

**Figure 2:**
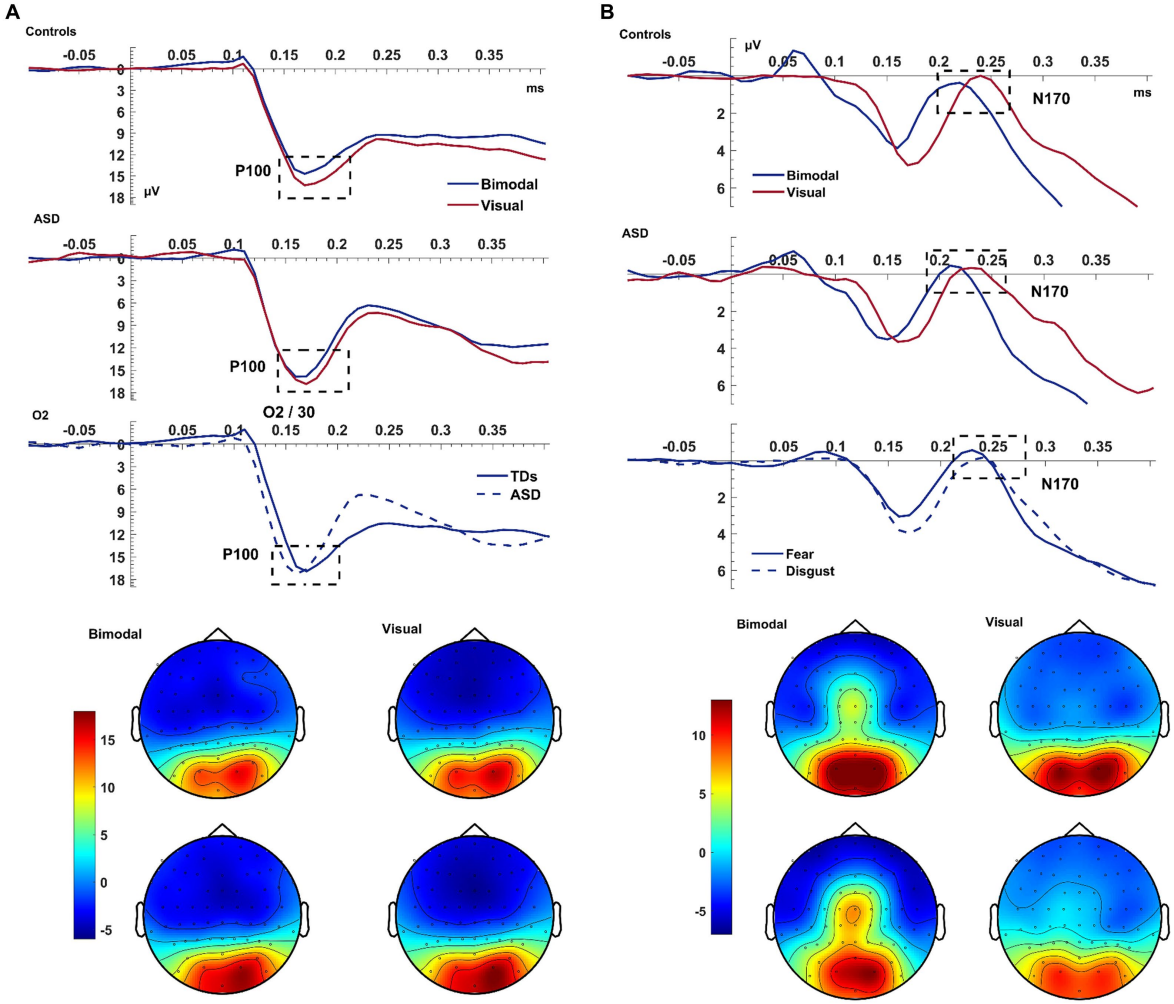
Visual P100 & N170. Panel **(A)** shows the average waveform (O1, Iz, O2) and topographies of the visual P100 component at the bimodal and visual conditions, for controls and ASD. The dashed box highlights the amplitude suppression and speeded latency of the component during the bimodal compared to the visual. The bottom row of the left panel shows a significant GROUP x ELECTRODE interaction with patients presenting faster latency than controls at electrode O2. Panel **(B)** shows the average waveform (T8, TP8, P2) and topographies of the visual N170 component at the bimodal and visual conditions, for controls and ASD. The bottom row of the panel shows the overall emotion effect with fear producing an overall increased amplitude. The Y-axis of the waveform represents activity in μV and the X-axis time in ms. The color bar represents the range of activity in μV for interpretation of the topographical maps.

In all subjects, the bimodal condition produced a smaller visual P100 amplitude compared to the visual condition [*F*_(1,38)_ = 10.589, *p* = 0.002, η_p_^2^ = 0.218; Figure 2]. There was a significant EMOTION*ELECTRODE interaction [*F*_(1,76)_ = 3.594, *p* = 0.032, η_p_^2^ = 0.086] with the post-hoc paired samples t-test revealed that the amplitude of P100 was larger for electrode O2 than for Iz and for trials of fear compared to disgust [*t*_(39)_ = 3.308, *p* = 0.002].

The latency of P100 was shorter following the bimodal compared to the visual stimuli [CONDITION: *F*_(1,38)_ = 5.60, *p* = 0.023, η_p_^2^ = 0.128] and for trials depicting fear compared to disgust [EMOTION: *F*_(1,38)_ = 5.537, *p* = 0.024, η_p_^2^ = 0.13]. The P100 was of shorter latency at electrode O2 compared to O1 [*F*_(2,76)_ = 5.949, *p* = 0.004, η_p_^2^ = 0.135], and, according to post-hoc paired samples t-test this difference was amplified for the emotion of fear compared to disgust [*t*_(39)_ = −2.335, *p* = 0.025; EMOTION*ELECTRODE: *F*_(2,76)_ = 3.622, *p* = 0.026, η_p_^2^ = 0.092]. Finally, the faster latency of the P100 as seen on electrode O2 was larger for patients than controls [*F*_(1,38)_ = 4.522, *p* = 0.040; ELECTRODE*GROUP: *F*_(2,76)_ = 5.673, *p* = 0.005, η_p_^2^ = 0.13; see Figure 2]. This interaction revealed a laterality effect for the P100 latency that was, according to post-hoc paired samples t-test performed separately for each group, present only in patients and not in controls [ASD: *t*_(14)_ = −2.576, *p* = 0.022; Controls: *t*_(24)_ = 0.153, *p* = 0.880].

To summarize the key findings, the bimodal condition produced a speed-up of the visual P100 but contrary to our expectations, the P100 amplitude decreased during the bimodal condition.

#### 3.2.2 Visual N170

The bimodal condition produced a larger N170 amplitude than the visual condition [*F*_(1,38)_ = 4.991, *p* = 0.031, η_p_^2^ = 0.116]. This difference was increased for trials of *fear* compared to *disgust*, with this EMOTION effect being greater for electrode TP8 compared to P2 [*t*_(39)_ = −3.255, *p* = 0.002] and P8 [*t*_(39)_ = −5.664, *p* < 0.001; ELECTRODE: *F*_(2,76)_ = 6.132, *p = 0*.004, η_p_^2^ = 0.14; CONDITION*ELECTRODE: *F*_(1.863,70.776)_ = 21.645, *p* < 0.001, η_p_^2^ = 0.36; CONDITION*EMOTION*ELECTRODE: *F*_(2,76)_ = 8.031, *p* = 0.001, η_p_^2^ = 0.17]. For the N170, however, IQ was associated with this effect on its amplitude [*F*_(1,38)_ = 4.488, *p* = 0.041]. After controlling for IQ, controls presented increased amplitude compared to patients, which was significant *only* for trials depicting fear during the visual condition and, according to post-hoc t-tests, was specific to electrode TP8 [*t*_(38)_ = −2.171, *p* = 0.036].

The bimodal condition also produced a shorter N170 latency than the visual condition with this being significant only for the emotion of fear [CONDITION: *F*_(1,38)_ = 5.2167, *p* = 0.029, η_p_^2^ = 0.120; EMOTION: *F*_(1,38)_ = 8.754, *p* = 0.005, η_p_^2^ = 0.187; CONDITION*EMOTION: *F*_(1,38)_ = 18.200, *p* < 0.001, η_p_^2^ = 0.324]. There was an additional interaction of EMOTION*ELECTRODE (p = 0.004) leading to a CONDITION*EMOTION*ELECTRODE*GROUP (p = 0.040) interaction which will not be further interpreted as it was driven purely by electrodes.

Similar to the P100, the bimodal condition again produced a speed-up of the N170 latency which was further shortened by the emotion of fear. In line with our hypothesis, the bimodal condition also produced an increased N170 amplitude and this was amplified for the emotion of fear; after controlling for IQ this was significantly higher for controls compared to autistic individuals and specific to the emotion of fear.

#### 3.2.3 Auditory N100

The bimodal condition produced an increased N100 amplitude compared to the auditory condition [*F*_(1,38)_ = 3.980, *p = 0*.053, η_p_^2^ = 0.095]. A CONDITION*EMOTION*GROUP interaction [*F*_(1,38)_ = 5.217, *p* = 0.028, η_p_^2^ = 0.121] further qualified this result. The difference between the bimodal and auditory conditions tended to be increased for trials of *disgust* rather than *fear* [EMOTION: *F*_(1,38)_ = 3.980, *p = 0*.053, η_p_^2^ = 0.095] and amplified for patients than controls. According to post-hoc ANOVAs (performed for each group separately), neither group presented an effect of CONDITION, EMOTION or a CONDITION*EMOTION interaction (all *p* = ns) indicating that this interaction was driven by groups presenting opposite CONDITION*EMOTION interactions.

#### 3.2.4 Auditory P200

**Figure 3:**
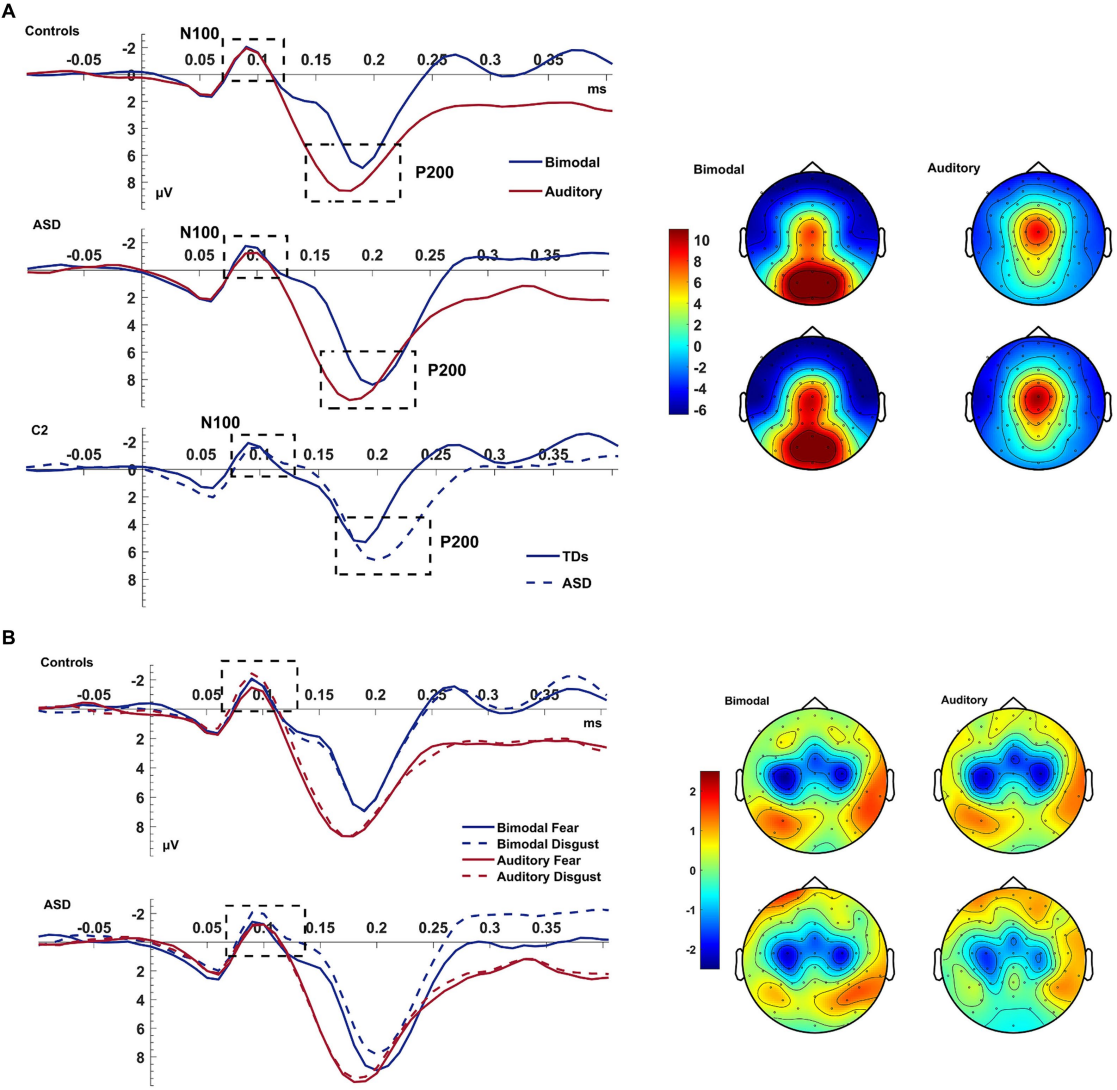
Auditory N100 & P200. Panel **(A)** shows the average waveform (C1, Cz, C2) of the auditory N100 and P200 components and the P200 topography at the bimodal and auditory conditions, for controls and ASD. The dashed box highlights the components. The P200 amplitude suppression of the component during the bimodal compared to the auditory condition was increased more for the ASD group than for controls who also showed an overall speeded P200 compared to the ASD group at electrode C2 (bottom row of left pane). Panel **(B)** shows the average waveform (C1, Cz, C2) of the auditory N100 for the CONDITION × EMOTION interaction and the topography at the bimodal and auditory conditions, for controls and ASD. The Y-axis of the waveform represents activity in μV and the X-axis time in ms. The color bar represents the range of activity in μV for interpretation of the topographical maps.

The P200 showed a decreased amplitude and a delayed latency during the bimodal compared to the auditory condition [CONDITION effects for amplitude: *F*_(1,38)_ = 12.043, *p* = 0.001, η_p_^2^ = 0.241; for latency: *F*_(1,38)_ = 29.364, *p* < 0.001, η_p_^2^ = 0.436; see Figure 3]. There was also an electrode effect [ELECTRODE: *F*_(1.769,67.239)_ = 3.306, *p* < 0.001, η_p_^2^ = 0.515]; planned contrasts revealed that, Cz was showing a larger amplitude than C1 (*p* < 0.001) and C2 (*p* < 0.001), and electrode C1 compared to C2 (*p* = 0.028). Controls presented again a trend for overall shorter latencies than patients; according to a one-way post-hoc ANOVA this difference was significant for electrode *C2* [GROUP effect for C2: *F*_(1,38)=_5.468, *p* = 0.025; ELECTRODE*GROUP: *F*_(1.681,63.886)_ = 3.360, *p = 0*.049, η_p_^2^ = 0.081; see Figure 3].

In summary, and contrary to our expectations, the auditory N100 was of higher amplitude during the bimodal condition which was increased for disgust—this was driven by the two groups showing opposing CONDITION*EMOTION interactions. The P200, on the other hand, showed the expected increased amplitude and delayed latency during the bimodal compared to the auditory condition.

#### 3.2.5 Late positive component

There were no significant main effects between the bimodal and visual conditions on the LPC mean area activity. There was, however, a significant crossover interaction with controls showing increased amplitude for fear compared to disgust and patients showing increased amplitude for disgust [EMOTION*GROUP*: F*_(1,38)_ = 5.154, *p* = 0.029, η_p_^2^ = 0.119]. This crossover interaction was further amplified for the visual condition [CONDITION* EMOTION*GROUP: *F*_(1,38)_ = 7.435, *p* = 0.010, η_p_^2^ = 0.164], and for electrode Poz [EMOTION*GROUP*ELECTRODE: *F*_(1,38)_ = 5.374, *p* = 0.026, η_p_^2^ = 0.124; CONDITION*EMOTION*GROUP*ELECTRODE: *F*_(1,38)_ = 4.987, *p* = 0.032, η_p_^2^ = 0.116]. According to post-hoc ANOVAs, these interactions were not significant (all *p* = ns) except for the CONDITION*EMOTION interaction which was present *only in patients* where the emotion of disgust produced an overall larger LPC deflection and this difference was further amplified for the visual compared to the bimodal condition [*F*_(1,14)_ = 6.646, *p* = 0.022, η_p_^2^ = 0.322].

With regards to the auditory condition, the LPC deflection was significantly larger under the bimodal compared to the auditory condition [CONDITION: *F*_(1,38)_ = 77.166, *p* < 0.001, η_p_^2^ = 0.670]. Furthermore, fear also produced a significantly larger amplitude than disgust and this difference was further amplified for patients compared to controls [EMOTION: *F*_(1,38)_ = 11.761, *p* < 0.001, η_p_^2^ = 0.236; EMOTION*GROUP: *F*_(1,38)_ = 8.467, *p* = 0.006, η_p_^2^ = 0.182]. According to a post-hoc ANOVA (performed for each group separately), the increase of the LPC deflection for the emotion of fear was significant *only for patients* [*F*_(1,14)_ = 15.062, *p* = 0.002, η_p_^2^ = 0.518] and not for controls (*p* = 0.669). There was an additional effect of electrode site [*F*_(1,38)_ = 30.611, *p* < 0.001, η_p_^2^ = 0.446] and CONDITION*ELECTRODE interaction [*F*_(1,38)_ = 27.856, *p* < 0.001, η_p_^2^ = 0.423] which will not be further interpreted as it was driven purely by electrodes.

### 3.3 Spatio-temporal evolution of MSI

Analysis revealed significant differences between the bimodal and sum conditions for the control group, with the bimodal condition showing both super-additivity and sub-additivity (this varied with topography) suggesting that MSI process is not the linear addition of the two signals. These effects started at 80 ms post-stimulus onset, with a central topography evolving to a more centro-parietal topography up to 120 ms. This MSI effect shifted to a more fronto-central and temporal topography up to 160 ms, changing to widespread topographical distribution involving both parieto-occipital and fronto-central areas; this effect lasted until 300 ms post-stimulus onset. Patients also showed some MSI effects, but these began about 50 ms later than controls (i.e., at 130 ms). From 200 ms onwards, this cluster shifted to a more centro-parietal but narrow topographical distribution (see Table 3 for statistics).

**Table 3:** Cluster-based permutation test statistics.

		Cluster statistic (df)	*p*-value	SD	CI range
**Controls**					
*Overall*	80–300 ms	1284.176 (24)	0.002	± 0.0014	± 0.0028
	90–300 ms	1210.812 (24)	0.002	± 0.0014	± 0.0028
*Fear*	100–290 ms	766.871 (24)	0.0002	± 0.0001	± 0.0003
	110–300 ms	−824.282 (24)	0.0002	± 0.0001	± 0.0003
*Disgust*	130–300 ms	944.042 (24)	0.0002	± 0.0001	± 0.0003
	130–300 ms	−1022.71 (24)	0.0002	± 0.0001	± 0.0003
**ASD**					
*Overall*	130–300 ms	−623.651 (14)	0.002	± 0.0014	± 0.0028
*Disgust*	150–240 ms	461.2 (14)	0.0064	± 0.0008	± 0.0016
	130–300 ms	−744.905 (14)	0.0008	± 0.003	± 0.0006

Cluster statistic denotes the *t*-value of the maximum cluster level statistic; SD, the standard deviation of the cluster statistic; and CI, Range the confidence interval.

**Figure 4:**
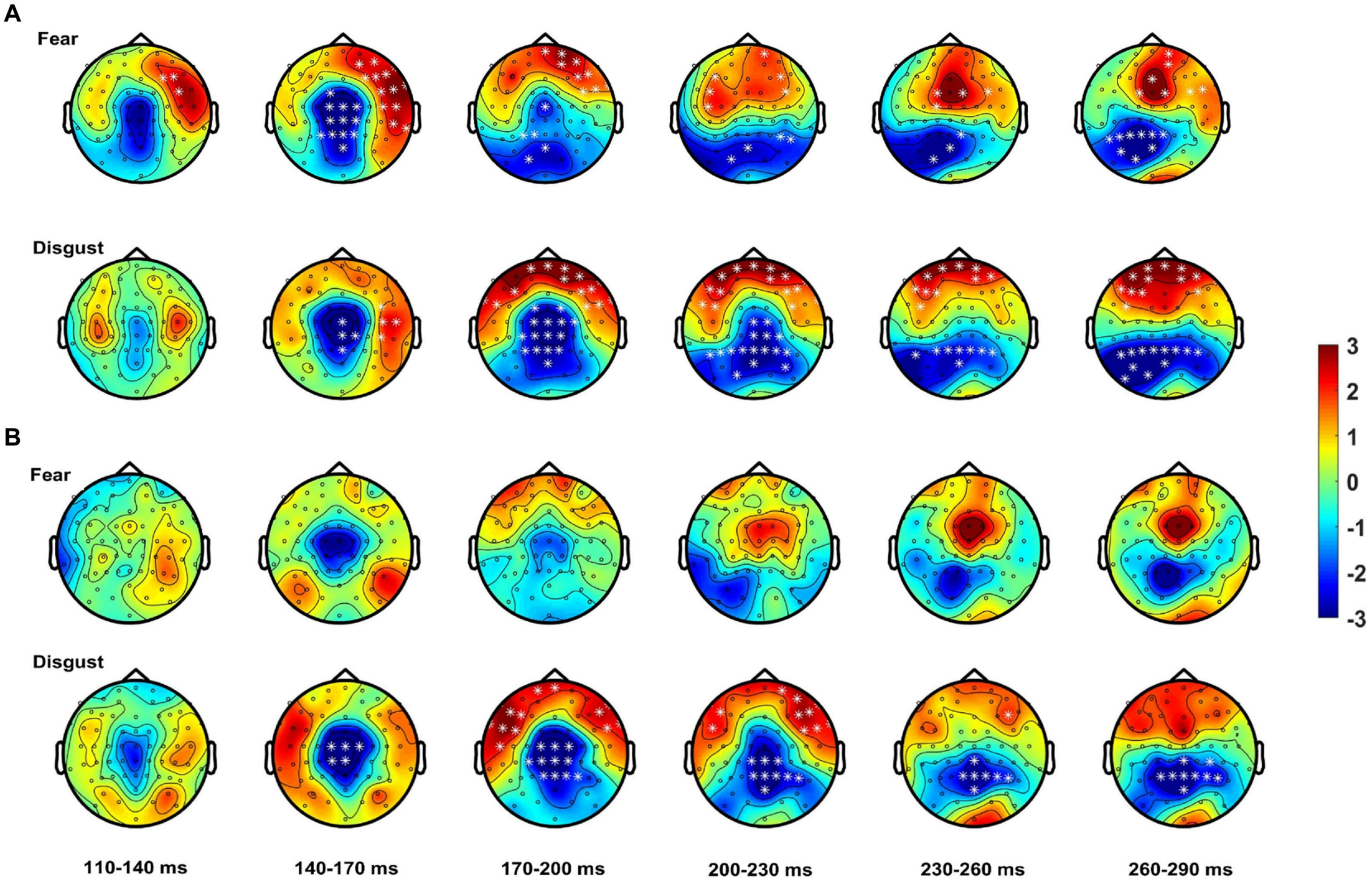
Spatio-temporal course of the MSI effect [Bimodal – (Audio + Visual)]. Topographies show the effect from 110 to 290 ms averaged over bins of 30 ms for **(A)** controls starting as early as 110 ms for fear (top row) and disgust (bottom row) from ~140 ms onwards. **(B)** Patients show no MSI effects for fear (top row) and weaker effects for disgust (bottom row). White asterisks highlight electrodes within clusters where the differences between the bimodal and sum (i.e., MSI) were significant.

When analysis was performed for each emotion separately, controls showed, for *fear*, MSI effects starting at 100 ms at frontal right-hemisphere areas, extending to central, frontal and bilateral temporal scalp areas. These effects lasted until 170 ms after stimulus onset and shifted to frontal and parieto-occipital areas until 300 ms post-stimulus. Notably, for the emotion of fear patients did not show any spatio-temporal effects (see Figure 4). For *disgust* relative to fear, controls showed a somewhat delayed MSI effect, starting at 130 ms at central and right-hemisphere temporal areas. From 160 ms onwards these effects extended to bilateral temporal, frontal and central scalp areas and evolved to occipital areas until 300 ms. Patients, this time, showed a similar but weaker MSI effect in terms of topographical distribution, starting as well at 130 ms at central scalp areas and including frontal electrodes from 160 ms onwards. This effect shifted from 230 ms to a topographically narrower central-parietal topography, reaching 300 ms (see Figure 4; Table 3).

In summary, these results confirm an early onset of MSI for controls with autistic individuals showing a delayed onset of MSI which, when analyzed for each emotion separately it was present only for the emotion of disgust.

## 4 Discussion

The present study set out to investigate the neural basis of MSI for emotion processing in autistic adolescents using highly ecologically valid stimuli (videos) expressing fear and disgust. We found the following main results. *With regards to the behavioral data*, we, firstly, observed that the bimodal condition produced speeded and more accurate responses compared to the auditory and visual conditions regardless of group. This facilitation of responses during the bimodal condition compared to the visual condition was amplified in the fear compared to disgust expression, while the speed-up seen in the bimodal compared to the auditory condition was amplified for disgust compared to fear. *Secondly*, autistic adolescents showed dampened or no MSI compared to controls, according to Miller’s RMI. In other words, although both groups showed a facilitation of RT, only in controls this was due to successful MSI.

With regards to the electrophysiological data, significant MSI effects were seen, firstly, in terms of shorter latencies of the visual P100 and N170 components and a delayed P200 latency and, secondly, in a suppressed visual P100 amplitude and an increased N170 amplitude. More importantly, MSI effects were seen in controls as early as 80 ms at several scalp areas with patients showing a delayed and spatially narrowed MSI effect, which was driven by the emotion of disgust only.

### 4.1 MSI effects and emotion differentiation

In accordance with previous studies (Collignon et al., 2008; Charbonneau et al., 2013) the bimodal presentation of the dynamic stimuli presenting fear and disgust produced significantly improved performance compared to the degraded unimodal conditions (Collignon et al., 2008; Stefanou et al., 2019). Regardless of group, responses to the bimodal condition were faster, more accurate and with decreased variance (SDRT) compared to the auditory and visual conditions. Additionally, *fear* also produced speeded and more accurate responses compared to disgust in the bimodal vs. visual contrast. Such a finding is in line with the dissociating effects of fear compared to disgust, with fear heightening attention and disgust dampening attention. In accordance with our previous study (Stefanou et al., 2019), the dissociating effects of fear and disgust extend to MSI with fear augmenting the speed-up of RT in the bimodal compared to the visual condition. This finding confirms that the attention-orienting effect of fear, due to its biological significance, does not occur only during unisensory processing but also during bimodal processing when voice and face convey the same emotion.

However, despite fear producing overall faster RT than disgust, the speed-up of responses in the bimodal condition, was amplified by the emotion of disgust. Although somewhat unexpected, such a finding could be related to the fact that in this case, the degraded channel in the bimodal condition was the auditory one, and not the visual one which is also considered to be the dominant channel (Colavita, 1974). This would be in accordance with the inverse effectiveness rule (Meredith and Stein, 1986; Stein et al., 1993); according to the inverse effectiveness rule first observed in animal studies, there is a stronger MSI benefit when one of the two signals of the bimodal stimulus is weak, while when one of the signals of the bimodal condition is strong, there is a reduced MSI. Findings following the inverse effectiveness rule have been reported in MSI studies with humans as well (Senkowski et al., 2011; Stefanou et al., 2019). Given that fear produced faster RT regardless of condition, the combination of these two augmenting effects could have possibly created a ceiling effect for the interaction of the redundant signal and fear.

MSI facilitation was further demonstrated by Miller’s RMI. Specifically, controls showed a significant MSI regardless of emotion, with fear facilitating a somewhat stronger MSI than disgust. Patients on the other hand showed MSI only for the emotion of fear, and not for disgust. These findings align with previously reported MSI deficits in autistic individuals, and further suggest that they can potentially compensate through the attentional mechanisms associated with fear. Despite the emotion of fear facilitating behavioral MSI in patients, this was decreased compared to controls. The above findings combined suggest that, regardless of emotion, ASD patients show behavioral MSI deficits replicating findings of previous studies (Brandwein et al., 2013; Charbonneau et al., 2013; Collignon et al., 2013). That patients show a behavioral MSI effect only for fear, according to Miller’s Model leads us to presume that any speed-up they showed in the conventional median RT analysis was caused by a *race* of the two signals in the bimodal condition and *not* by the integration of the audiovisual signals. Indeed, conventional RT analysis cannot definitively argue whether MSI has occurred or if the speed is a result of statistical facilitation, whereas Miller’s RMI provides an indication of an underlying coactivation mechanism (Colonius and Diederich, 2006), indicating thus more reliably whether MSI occurred or not. These findings also validate the dissociating effects that fear and disgust have on attention and by consequence to MSI, with the enhancing effects of fear on MSI being observed in the autistic group as well and suggesting it can facilitate MSI even in individuals with MSI deficits. Such a conclusion is reinforced by studies reporting that attention improves MSI (Magnée et al., 2011; Fernández et al., 2015) and that ASD patients are capable of MSI if they actively attend to stimuli (Russo et al., 2010) and potentially compensate for MSI deficits via later attentional processes (Stefanou et al., 2020), in this case by the attention orienting effect of fear (Susskind et al., 2008; Krusemark and Li, 2011).

### 4.2 MSI in electrophysiological responses

In line with previous studies (Brefczynski-Lewis et al., 2009; Jessen and Kotz, 2011), the visual P100 and N170 components peaked significantly earlier during the bimodal compared to the visual condition. Unexpectedly, ASD patients, regardless of condition, presented a speeded appearance of the P100 compared to controls. The speed-up of the bimodal N170 was increased only for fear, potentially due to the aforementioned attentional effects of fear and disgust (Susskind et al., 2008).

In contrast to our previous results and general findings of super-additivity both in EEG and fMRI studies (Pourtois et al., 2005; Brandwein et al., 2011; Stefanou et al., 2019; for a review, see Campanella and Belin, 2007), we found decreased amplitude of the visual P100 in the bimodal compared to the visual condition. Despite the visual P100 being linked to the facilitation of sensory processing for stimuli at attended locations (Luck et al., 1990; Mangun, 1995; Hillyard et al., 1998), this amplitude suppression does not necessarily imply decreased facilitation of sensory processing in the bimodal condition. Given that we compared the visual with the bimodal condition when the visual signal was degraded (see Methods), we cannot be certain how this degradation, in combination with the MSI effect, has manipulated the bimodal P100 amplitude. However, fear once more produced increased amplitude for the P100 compared to disgust corroborating previous statements of the diverging effects of fear and disgust.

We further report an overall MSI-related increase of the N170 amplitude which was greater for fear than disgust, in agreement with our previous results (Stefanou et al., 2019) but see also (Brefczynski-Lewis et al., 2009, reporting sub-additive effects of MSI, i.e., reduced amplitude on the N170). This N170 amplitude increase suggests that the different effects fear and disgust have on attention, with fear capturing attention (Susskind et al., 2008) enhanced the processing of the stimuli facilitating MSI. Although this increase of the N170 amplitude contradicts the decreased amplitude during the bimodal condition found by Brefczynski-Lewis et al. (2009), this could result from the fact that our stimuli depicted emotions of real persons whereas their stimuli were non-emotional portrays of synthetic faces. Additionally, fear, as compared to disgust, increases the negativity of the N170 (Batty and Taylor, 2003; Almeida et al., 2016) since it presumably elicits a unique response compared to other emotions. Thus, the fear-related increase of the N170 amplitude suggests greater facilitation of sensory processing of fear compared to disgust, for both uni- and multisensory processing.

With regards to the auditory components, the bimodal condition produced an increase of the N100 deflection, and the emotion of disgust further amplified this effect. The increase of the N100 amplitude is in contrast with other studies using ecologically valid stimuli (Stekelenburg and Vroomen, 2007; Jessen and Kotz, 2011) reporting a reduced N100 amplitude during bimodal conditions. However, post-hoc analyses revealed no modulation of the N100 amplitude, neither by condition nor emotions and no interaction of the two factors. This amplitude increase is, at least topographically, elicited by disgust in the ASD group and by fear in controls. If these are replicated by future studies, it could suggest that the attention-orienting and attention-diverting effects of fear and disgust operated inversely in patients. Such a finding is in line with reports of altered emotion perception in ASD (Stewart et al., 2013; Uljarevic and Hamilton, 2013; Globerson et al., 2015; Bestelmeyer et al., 2018). This finding further argues this deficit cannot be permeated by biologically significant events, at least not at its neurophysiological substrates. This differentiation between controls and patients in MSI processing can be seen by a delayed N100 latency in the bimodal condition, which is again driven by patients. Given that the auditory N100 is an initial orienting response (O’Connor, 2012) the delayed latencies observed in patients compared to controls point to decreased attention orienting, at least at initial processing stages, and an overall slower sensory processing regardless of any MSI-related effects.

As with our previous findings (Stefanou et al., 2020), we report a delayed P200 latency at the bimodal compared to the auditory condition. Given that *improved* performance and discriminability (Rif et al., 1991; Lijffijt et al., 2009) are linked to a *delayed* auditory P200, and since there was an overall P200 delay in ASD patients compared to controls, our results suggest greater attentional effort. This attentional effort of the ASD group, as indexed by the overall delayed P200 and a larger MSI-related P200 delay compared to controls, could possibly point to the recruitment of attentional mechanisms in order to compensate for auditory processing and MSI-related deficits. The bimodal condition also produced a suppression of the auditory P200 which could be a delayed integrative effect not seen in the auditory N100. Further support of a potential increased attentional effort, to compensate for early sensory deficits can be seen from the increased LPC amplitude that patients show compared to controls for the emotion of fear. Indeed, the literature suggests that the LPC is not only related to emotion processing but its amplitude additionally increases to biologically related stimuli such as threat (Hajcak et al., 2012) while previous studies have revealed increased LPC amplitudes for emotional stimuli with it being increased when these stimuli are targets (Schindler and Kissler, 2016).

### 4.3 Spatio-temporal evolution of MSI

The aforementioned MSI effects were confirmed by the cluster-based permutation tests, which contrasted the bimodal condition with to the sum of the two unimodal conditions. Early MSI effects were readily observable in controls, with a topographically widespread distribution, starting as early as 80 ms and lasting up to 300 ms post-stimulus onset. These findings are in line with our previous results (Stefanou et al., 2020) as well as with other studies (Russo et al., 2010; Brandwein et al., 2013) suggesting that, MSI in healthy individuals occurs before basic sensory processing does (e.g., P100). Furthermore, controls presented similar MSI effects for both the emotions of fear and disgust. Still, the MSI effects for fear started earlier than those for disgust corroborating that fear not only enhances attention due to its biological purpose (Susskind et al., 2008) but that its effects facilitate complex processes such as MSI.

By contrast, patients showed a delayed overall MSI effect compared to controls. This effect started at 130 ms and showed a restrained topographical distribution suggesting that autistic individuals in our study present altered MSI. Furthermore, patients with ASD showed MSI only for the emotion of disgust which presumably elicited the overall MSI effect.

The overall delayed MSI effects seen in patients compared to controls, suggests altered MSI for which they compensate at later processing stages possibly through attentional mechanisms (Koelewijn et al., 2010; Stefanou et al., 2020). The latency where we first observe this MSI effect (130 ms) in patients, along with the MSI-related P200 delay suggests an altered MSI in ASD during early processing stages, which is compensated for at later processing stages.

The present study has limitations that narrow the generalisability of our results. Firstly, the sample size was overall rather small reducing the statistical power of the study. Secondly, we degraded the unimodal conditions to account for unisensory dominance that would reduce any MSI facilitation (see Collignon et al., 2008; Stefanou et al., 2019). However, we cannot be certain how this may have affected the electrophysiological results, given that the components under investigation are exogenous components representing the facilitation of sensory processing. During the preliminary noise threshold task, patients differed compared to controls only for the visual noise threshold which may have also affected the results. However, the main purpose of this degradation was to ensure that all participants started the main experiment with 80% accuracy for the degraded unimodal conditions, suggesting that the reported differences between the bimodal and unimodal conditions are due to MSI differences and not due to noise sensitivity levels of each group. Thirdly, because of previous literature reporting altered MSI in ADHD and individuals with ADHD traits (see Panagiotidi et al., 2017) we did not recruit participants with comorbid ADHD. Therefore, our results would not be representative to the subgroups of autistic individuals with comorbid ADHD. Furthermore, although our age range is rather narrow, evidence from previous research reveals that key differences in MSI facilitation are identified between children before the age of 10 years old, and older than 11 years old (Brandwein et al., 2013). It has been further suggested that MSI reaches maturation levels by the age of 14 years old (Brandwein et al., 2011) suggesting that our age-range should in principle suffice to investigate MSI in the adolescence period.

However, to conclude, the results of the present study point to altered emotion recognition and altered MSI in ASD. Such a finding would be in line with theories of disrupted connectivity (for reviews, see Belmonte et al., 2004; Hughes, 2007; Hornix et al., 2019) as this altered long-range connectivity may underlie MSI deficits due to an insufficient synchronization between the involved areas (Martínez-Sanchis, 2014). We present evidence of altered MSI in ASD which, behaviorally, can be masked by biologically significant events such as fear via attention orienting. Such attention-driven compensatory mechanisms, as seen in patients, are reinforced by the electrophysiological data showing a similar compensatory mechanism in the form of *attentional effort* and not *attentional orienting* driven by fear.

## Data availability statement

Datasets generated for this manuscript are available from the corresponding author upon request.

## Ethics statement

The studies involving humans were approved by the Ethics Committee of the Albert Ludwigs-University of Freiburg (ethics vote no. 238/15). The studies were conducted in accordance with the local legislation and institutional requirements. Written informed consent for participation in this study was provided by the participants and their legal guardians/next of kin.

## Author contributions

MES: Writing – review & editing, Investigation, Writing – original draft, Visualization, Software, Formal analysis, Data curation, Conceptualization. ND: Writing – review & editing, Software, Conceptualization. PB: Writing – review & editing. MB: Writing – review & editing. NS: Writing – review & editing, Conceptualization. CK: Writing – review & editing, Supervision, Conceptualization.
