# Supplementary material for: The dissociating effects of fear and disgust on multisensory integration in autism: evidence from evoked potentials
Source: Front Neurosci. 2024 Aug 5;18:1390696. doi: 10.3389/fnins.2024.1390696 (PMC11330835; doi:10.3389/fnins.2024.1390696)
Supplement: Supplementary file 1 [file Table_1.DOCX]

**Supplementary Table 1.** Statistical results of behavioural responses’ comparisons for median reaction times (RT), the standard deviation of reaction times (SDRT) and accuracy for the two mixed model ANOVAs (see Methods). Auditory vs AV_A indicates the contrast between the auditory and audiovisual condition degraded with auditory noise and the Visual vs AV_V the contrast between visual and audiovisual condition degraded with visual noise.

|  | | **Mixed model ANOVA table** | | | | | | | | | | | | | | |
| --- | --- | --- | --- | --- | --- | --- | --- | --- | --- | --- | --- | --- | --- | --- | --- | --- |
|  |  | **Median RT (ms)** | | | | **SDRT (ms)** | | | | | **Accuracy (%)** | | | | | |
| **Auditory vs AV_A** | | **F_(df)_** | ***p*** | **η_p_^2^** | | **F_(df)_** | ***p*** | **η_p_^2^** | | | **F_(df)_** | | | ***p*** | | **η_p_^2^** |
|  | Group | F_(1,38 )_ = 0.11 | .740 | .003 | | F_(1,38 )_ = 0.51 | .480 | .013 | | | F_(1,38 )_ = 1.19 | | | .283 | | .030 |
|  | Condition | F_(1,38 )_ = 90.03 | **<.001** | .703 | | F_(1,38)_ = 56.43 | **<.001** | .598 | | | F_(1,38)_ = 60.06 | | | **<.001** | | .612 |
|  | Group x Condition | F_(1,38 )_ = 1.99 | .166 | .050 | | F_(1,38 )_ = 0.16 | .689 | .004 | | | F_(1,38 )_ = 0.29 | | | .593 | | .008 |
|  | Emotion | F_(1,38 )_ = 7.97 | **.008** | .173 | | F_(1,38 )_ = 0.06 | .814 | .001 | | | F_(1,38 )_ = 0.01 | | | .941 | | 0 |
|  | Emotion x Condition | F_(1,38 )_ = 6.5 | **.015** | .146 | | F_(1,38 )_ = 1.47 | .232 | .037 | | | F_(1,38 )_ = 4.87 | | | **.033** | | .114 |
|  | Group x Emotion | F_(1,38 )_ = 0.15 | .704 | .004 | | F_(1,38 )_ = 0.31 | .583 | | .008 | | | F_(1,38 )_ = 0 | | .991 | | 0 |
|  | Group x Emotion x Condition | F_(1,38 )_ = .22 | .642 | .006 | | F_(1,38 )_ = 0.002 | .961 | | 0 | | | F_(1,38 )_ = 0.002 | | .966 | | 0 |
|  | |  | | | | | | | | | | | | | | |
| **Visual vs AV_V** | |  | | | | | | | | | | | | | | |
|  | Group | F_(1,38 )_ = 0.90 | .348 | | .023 | F_(1,38 )_ = 0.401 | .530 | | | 0.010 | | | F_(1,38 )_ = 1.28 | | .265 | .033 |
|  | Condition | F_(1,38 )_ = 31.51 | **<.001** | | .618 | F_(1,38)_ = 9.78 | **.003** | | | .205 | | | F_(1,38)_ = 34.87 | | **<.001** | .478 |
|  | Group x Condition | F_(1,38 )_ = 0.01 | .926 | | 0 | F_(1,38 )_ = 0.1 | .750 | | | 0.03 | | | F_(1,38 )_ = 0.99 | | .325 | .025 |
|  | Emotion | F_(1,38 )_ = 27.96 | **<.001** | | .424 | F_(1,38 )_ = 1.07 | .308 | | | .027 | | | F_(1,38 )_ = 7.29 | | **.010** | .161 |
|  | Emotion x Condition | F_(1,38 )_ = 8.39 | **.006** | | .181 | F_(1,38 )_ = 0.307 | .583 | | | .008 | | | F_(1,38 )_ = 6.462 | | **.015** | .145 |
|  | Group x Emotion | F_(1,38 )_ = 2.60 | .115 | | .064 | F_(1,38 )_ = 0.77 | .386 | | | .020 | | | F_(1,38 )_ = 0.270 | | .606 | .007 |
|  | Group x Emotion x Condition | F_(1,38 )_ = .01 | .947 | | 0 | F_(1,38 )_ = 0.81 | .373 | | | .021 | | | F_(1,38 )_ = 0.78 | | .383 | .020 |
